# *Anaplasma phagocytophilum* evolves in geographical and biotic niches of vertebrates and ticks

**DOI:** 10.1186/s13071-019-3583-8

**Published:** 2019-06-28

**Authors:** Ryanne I. Jaarsma, Hein Sprong, Katsuhisa Takumi, Maria Kazimirova, Cornelia Silaghi, Atle Mysterud, Ivo Rudolf, Relja Beck, Gábor Földvári, Laura Tomassone, Margit Groenevelt, Reinard R. Everts, Jolianne M. Rijks, Frauke Ecke, Birger Hörnfeldt, David Modrý, Karolina Majerová, Jan Votýpka, Agustín Estrada-Peña

**Affiliations:** 1 Centre for Infectious Disease Control (CIb), National Institute for Public Health and the Environment (RIVM), Bilthoven, The Netherlands; 2 Laboratory of Entomology, Wageningen University, Wageningen, The Netherlands; 3 Institute of Zoology, Slovak Academy of Sciences, Bratislava, Slovakia; 4 Friedrich-Loeffler-Institut, Federal Research Institute for Animal Health, Greifswald-Insel Riems, Germany; 5 Centre for Ecological and Evolutionary Synthesis (CEES), Department of Biosciences, University of Oslo, P.O. Box 1066, Blindern, 0316 Oslo, Norway; 6 Institute of Vertebrate Biology, v.v.i, Medical Zoology Laboratory, Academy of Sciences of the Czech Republic, Brno, Czech Republic; 7 Department for Bacteriology and Parasitology, Croatian Veterinary Institute, Savska Cesta 143, Zagreb, Croatia; 8 Department of Parasitology and Zoology, University of Veterinary Medicine Budapest, Budapest, Hungary; 9 Evolutionary Systems Research Group, Centre for Ecological Research, Hungarian Academy of Sciences, Tihany, Hungary; 10 Department of Veterinary Sciences, University of Turin, Via L. da Vinci 44, Grugliasco, 10095 Turin, Italy; 11 Diergeneeskundig Centrum Zuid-Oost Drenthe, Coevorden, The Netherlands; 12 Dutch Wildlife Health Centre, Utrecht University, Utrecht, The Netherlands; 13 Department of Wildlife, Fish, and Environmental Studies, Swedish University of Agricultural Sciences, Umeå, Sweden; 14 Department of Pathology and Parasitology, Faculty of Veterinary Medicine, University of Veterinary and Pharmaceutical Sciences, Brno, Czech Republic; 15 Biology Centre, Institute of Parasitology, Czech Academy of Sciences, České Budějovice, Czech Republic; 16 Department of Parasitology, Faculty of Science, Charles University, Prague, Czech Republic; 17 Department of Parasitology, Faculty of Veterinary Medicine, University of Zaragoza, Zaragoza, Spain; 18 Emerging Zoonoses Research Group, Instituto Agroalimentario de Aragón (IA2), Zaragoza, Spain

**Keywords:** *Anaplasma phagocytophilum*, Ticks, Ixodidae, Molecular epidemiology, Transmission dynamics, Network analysis

## Abstract

**Background:**

*Anaplasma phagocytophilum* is currently regarded as a single species. However, molecular studies indicate that it can be subdivided into ecotypes, each with distinct but overlapping transmission cycle. Here, we evaluate the interactions between and within clusters of haplotypes of the bacterium isolated from vertebrates and ticks, using phylogenetic and network-based methods.

**Methods:**

The presence of *A. phagocytophilum* DNA was determined in ticks and vertebrate tissue samples. A fragment of the *groEl* gene was amplified and sequenced from qPCR-positive lysates. Additional *groEl* sequences from ticks and vertebrate reservoirs were obtained from GenBank and through literature searches, resulting in a dataset consisting of 1623 *A. phagocytophilum* field isolates. Phylogenetic analyses were used to infer clusters of haplotypes and to assess phylogenetic clustering of *A. phagocytophilum* in vertebrates or ticks. Network-based methods were used to resolve host-vector interactions and their relative importance in the segregating communities of haplotypes.

**Results:**

Phylogenetic analyses resulted in 199 haplotypes within eight network-derived clusters, which were allocated to four ecotypes. The interactions of haplotypes between ticks, vertebrates and geographical origin, were visualized and quantified from networks. A high number of haplotypes were recorded in the tick *Ixodes ricinus*. Communities of *A. phagocytophilum* recorded from Korea, Japan, Far Eastern Russia, as well as those associated with rodents had no links with the larger set of isolates associated with *I. ricinus*, suggesting different evolutionary pressures. Rodents appeared to have a range of haplotypes associated with either *Ixodes trianguliceps* or *Ixodes persulcatus* and *Ixodes pavlovskyi*. Haplotypes found in rodents in Russia had low similarities with those recorded in rodents in other regions and shaped separate communities.

**Conclusions:**

The *groEl* gene fragment of *A. phagocytophilum* provides information about spatial segregation and associations of haplotypes to particular vector-host interactions. Further research is needed to understand the circulation of this bacterium in the gap between Europe and Asia before the overview of the speciation features of this bacterium is complete. Environmental traits may also play a role in the evolution of *A. phagocytophilum* in ecotypes through yet unknown relationships.

**Electronic supplementary material:**

The online version of this article (10.1186/s13071-019-3583-8) contains supplementary material, which is available to authorized users.

## Background

Communities of organisms co-evolve across time and space [1–4]. Recent studies have stressed the need to capture the ecological relationships of large sets of interacting species [5, 6]. Many tick-borne pathogens have a considerable impact on human, livestock and wildlife health. The different species of ticks and associated pathogens differ largely in the level of specialization for different wildlife hosts. Due to this complexity, the understanding of transmission cycles of most pathogens and the resulting phylogeny is still rudimentary. The application of graph analysis is a promising approach to understand the coevolution of the foci of ticks and associated pathogens [5, 7]. Properties of network analysis allow for the visualization and quantification of the peculiarities of their ecological relationships [8], revealing more or less distinct and/or nested subgroups of interacting organisms [9]. Modularity is a key element in these constructs, characterizing the degree of interactions of organisms among themselves and with other members of the network. For the interacting triad of ticks, pathogens and their vertebrate hosts, a high clustering and nestedness seem to be the rule rather than the exception [10]: some organisms interact more frequently among them than with other species, generating nested “communities” when the complete set of interactions is analyzed.

*Anaplasma phagocytophilum* is the etiological agent of human granulocytic anaplasmosis (HGA), and tick-borne fever in domesticated animals [11–13]. Although a wide range of wildlife species can be infected with *A. phagocytophilum*, the impact of these infections on wildlife health is unclear [14]. The main vectors of *A. phagocytophilum* are ticks of the *Ixodes ricinus* complex: *Ixodes ricinus* in Europe, *Ixodes persulcatus* in eastern Europe and East Asia, and *Ixodes scapularis* and *Ixodes pacificus* in North America, although several other *Ixodes* species have been implicated in maintaining *A. phagocytophilum* in enzootic cycles as well [12, 13, 15]. The transmission dynamics of A. phagocytophilum predominantly rely on horizontal transmission between ticks and vertebrate hosts and on transstadial transmission in its vectors. While its vertical transmission (transovarial) has only been documented for *Dermacentor albipictus* in laboratory conditions [16], no conclusive evidence of such a route has been reported in *Ixodes* ticks. Therefore, *A. phagocytophilum* is exposed to the evolutionary pressures of complex interactions among the vertebrate reservoirs and its vectors, which are instrumental in shaping the underlying tapestry of the genetic constellation of *A. phagocytophilum*.

The role of wildlife species in the circulation of *A. phagocytophilum* is yet to be clearly determined, but several species of wild ruminants are thought to be important reservoirs [17]. Free ranging ruminants, such as the roe deer (*Capreolus capreolus*) in Europe [18] and the white-tailed deer (*Odocoileus virginianus*) in America [19], also largely contribute to the propagation of the ticks. Small mammals, on the other hand, contribute more to the feeding of immature stages of *I. ricinus* species [18–20]. These animals also harbour nidicolous ticks, such as *Ixodes acuminatus* and *Ixodes trianguliceps*, which have been implicated to circulate *A. phagocytophilum* in cryptic cycles [21]. Domesticated animals sometimes act as super-spreaders of ticks, and maybe also of *A. phagocytophilum*, since they boost amplification cycles by feeding large numbers of ticks [22]. Genetic analyses could help to further elucidate the pathogenicity and zoonotic potential of particular *A. phagocytophilum* isolates, as well as the direction of spill-over between livestock and wildlife.

Since its reclassification in 2001 [23], *A. phagocytophilum* is viewed as a single species based on genetic analyses of *16S* rRNA, *groEL* and surface protein genes. This taxon includes what formerly had been recorded as *Ehrlichia equi*, the etiological agent of equine ehrlichiosis, the unnamed causative agent of human granulocytic ehrlichiosis, and *Ehrlichia phagocytophila*. However, the circulating variants of *A. phagocytophilum* do not equally infect different hosts or result in the same clinical picture. While *16S* rRNA is valuable for rough phylogenetic reconstruction, more variable genes such as *ankA*, *msp4* and *groEL* heat-shock operons are preferred to capture strains and population structure [15, 24, 25]. Two studies proposed the existence of four clusters of *A. phagocytophilum* strains based on the *ankA* gene [24, 26]. Both studies concur in the clear separation of variants recorded in humans and roe deer. A distinct lineage associated with rodents in Europe has also been described, which is believed to be vectored by *I. trianguliceps* [21]. Furthermore, one multi-locus approach [27] showed that strains from roe deer, voles and shrews did not fall into the same clonal complex as the variants infecting humans, dogs and horses, while wild boars (*Sus scrofa*) and hedgehogs (*Erinaceus europaeus*) could be reservoirs for a zoonotic *A. phagocytophilum* variant. Red deer (*Cervus elaphus*) have also been suspected of harbouring zoonotic types [28], as well as pathogenic variants of domestic ruminants [26, 27, 29, 30]. A previous study [31] supported these findings, and identified four different ecotypes in Europe based on a fragment of the *groEl* gene. The study revealed that the zoonotic ecotype could be linked to a multitude of hosts, but did not cluster with the bird, rodent or roe deer ecotypes.

In this study, we follow the working theory of Jahfari et al. [31] that an ecotype is a cluster of genetically similar *A. phagocytophilum* isolates based on *groEL* sequences. We expand previous concepts exploring the relationships among haplotypes of *A. phagocytophilum*, its reservoirs and the ticks involved in its circulation. By unifying methods from the fields of molecular biology, phylogenetics, and network theory, we test whether and quantify how *A. phagocytophilum* segregates into nested, interconnected networks of ticks and vertebrates, quantifying the levels of hierarchy embedded in the large background. We apply indices of modularity and centrality of the network, together with unambiguous measurements of phylogenetic clustering, to the largest available dataset of haplotypes of the bacterium to show how the ecological relationships of *A. phagocytophilum* emerge into a robust, nested, and connected epidemiological structure, which the bacterium exploits to diversify, spread into new niches and evolve along different strains.

## Methods

### Collection of *A. phagocytophilum* field isolates

The collection of *A. phagocytophilum* field isolates predominantly relied on convenience sampling from numerous previous or ongoing studies in Europe [32–49]. Ticks and tissue samples from vertebrates were stored below − 20 °C before further processing in the laboratory. All *A. phagocytophilum* field isolates are described in Additional file 1: Table S1.

### DNA extraction and sequencing

DNA from vertebrate samples and engorged ticks was extracted in different laboratories using various techniques [32–48]. DNA from tissues and engorged ticks which were specifically analysed for this study were extracted with the DNeasy^®^ Blood & Tissue kit (Qiagen, Hilden, Germany) as per the manufacturer’s instructions. Ticks from the vegetation were lysed with ammonium hydroxide [50]. These samples were screened for the presence of *A. phagocytophilum* DNA with a real-time polymerase chain reaction (qPCR) targeting a 77-bp portion of the *msp2* gene [51]. Amplification of the qPCR-positive samples was performed, targeting a 530-bp fragment of the *groEL* gene of *A. phagocytophilum* following published methods [52]. The PCR-products were analyzed with gel electrophoreses on a 1.5% agarose gel and coloured with SYBR™ Gold Nucleic Acid Gel Stain (Invitrogen, Carlsbad, CA, USA). When the initial PCR did not result in a visible product, a nested PCR was performed using the primers ApNest-F (5′-GTG GAA TTT GAA AAT CCA TAC-3′) and ApNest-R (5′-GTC CTG CTA GCT ATG CTT TC-3′). The PCR program had a pre-incubation step of 95 °C for 15 min, followed by 40 cycles of 30 s at 94 °C, 30 s at 55 °C and 40 s at 72 °C. Final extension was performed using a 10 min step at 72 °C. This nested PCR results in a 366-bp fragment. The PCR products were cleaned with ExoSAP-IT™ PCR Product Cleanup Reagent (Applied Biosystems, Foster City, CA, USA) and sequenced by BaseClear (Leiden, Netherlands). The chromatographs of the sequences were analyzed and the primer sites were trimmed in Bionumerics v.7.6 (Applied Maths, Sint-Martens-Latem, Belgium).

### Compilation of the molecular epidemiological dataset

We compiled a dataset with the *A. phagocytophilum* isolates that had a *groEL* DNA sequence, the geographical origin (country) and information on vertebrate/tick species from which the isolate originated [31]. A search for additional *A. phagocytophilum* isolates with an explicit statement of the geographical origin (country) and vertebrate/tick from which the isolate originated was performed in the Entrez Nucleotide Database. Isolates that did not originate from field or case studies, or lacked the minimum required epidemiological information were excluded. Only *A. phagocytophilum* isolates which contained the DNA fragment from 655 to 1020 (366) bp of the *groEL* open reading frame, using GenBank entry CP015376 as a reference, were included in the initial dataset. The complete dataset with DNA sequences is included in Additional file 1: Table S1 and consists of 1992 field isolates, including the samples obtained from GenBank. While the size of this fragment had previously been used to identify ecotypes [31], a smaller subset of isolates with *groEL* sequences that spanned a longer fragment with a higher genetic resolution (LF), from 589 to 1118 (530) bp, was extracted for further analyses. After this selection, the dataset consisted of 1623 field isolates. We acknowledge that the grouping according to large administrative divisions (countries) is too rough to describe the fine geographical structure of segregation of *A. phagocytophilum*. While data on provinces or localities of collection were available for some samples, their use (instead of the country) reduced the available dataset by about 50%. We thus decided to keep the name of the country as the only geographical indicator, to better exploit a longer list of available data.

### Haplotype and cluster delineation

A haplotype was defined here as a group of *A. phagocytophilum* isolates in the dataset in which all members shared an identical 530-bp fragment of the *groEL* gene. For this, a multiple alignment based UPGMA tree was generated in BioNumerics with *groEl* sequences that had been extracted from the dataset. Molecularly similar haplotypes (clusters) were identified according to published methods [31].

### Vertebrate and arthropod haplotype distributions between clusters

We tested the possibility of over-representation of a cluster among the isolates using a multinomial model in which an isolate from a single species is evenly associated across all eight clusters, i.e. with the probability of 1/8 per cluster. The Monte Carlo method was used to estimate the probability (*P*-value) that the number of most numerous clusters in a random realization from the multinomial is equal to or greater than the observed maximum among the isolates. These calculations were performed in Mathematica v.11.3 [53]. A probability of less than 0.05 was considered significant support for selective distribution.

### Building associations among ticks-reservoirs-haplotypes: associations of clusters with carriers and geography

Once the list of haplotypes of *A. phagocytophilum* isolates was completed with data about the species of vertebrate or tick and the country of origin, a network capturing interactions was built. While molecular methods delineate the evolution of the target sequence of the bacterium, a network records how the strains of *A. phagocytophilum* segregate throughout the ticks and the vertebrates involved in its circulation. In our application, a “record” is a pairwise *A. phagocytophilum*-tick/vertebrate combination at a single geographical site. Each time a haplotype was found linked to either a vertebrate or a tick, a link was drawn, matching haplotype-vertebrate or haplotype-tick for a given country. The complete network includes the number of times each haplotype was found in each species of vertebrate or tick plus the country. In our application, nodes represent “carriers” (ticks or vertebrates) that are linked to a “cargo” (a haplotype of *A. phagocytophilum*) in a given country.

We examined several hypotheses regarding the relationships among the partners of the network. Modularity resolves communities of organisms that interact more among themselves than with other members of the network. We used the Louvain algorithm for calculating modularity, as integrated in the software Gephi v.0.92 [54], detecting communities of haplotypes of *A. phagocytophilum* and the carriers to which they are associated. Modularity resolves the compartmentalization of the network, displaying subnetworks of interacting entities separated from other subnetworks. These nested structures provide information about which elements (carriers, cargo) should be considered a “sub-part” of another larger network element, which is a major indication of clusters of haplotypes being derived from another, probably larger, cluster of samples. We used the algorithm provided by Bastolla et al. [55] for calculation of nestedness. We also calculated the articulation points of these subnetworks, using the package *igraph* [56] of the R programming environment [57]. Articulation points are the nodes linking two or more different communities, whose removal increases the number of isolated sub-networks.

We already demonstrated that the structure of the networks in which tick-transmitted pathogens circulate correlates with critical scales of connectivity between ticks and hosts and that these relationships can be described based on their centrality indices. Centrality measures in ecological networks indicate the presence of “high-ranking nodes in the network that have significantly higher-than-average connectivity” [58]. Identifying the most central nodes was addressed using the betweenness centrality (BNC) defined as the number of shortest paths between pairs of nodes that pass throughout a given node, and the PageRank (PR) defined as the number and quality of links to a node to estimate how important the node is. The underlying assumption is that more important nodes are likely to receive more links from other nodes. We tested whether these high centrality nodes exist in each community, and how they are inter-related. A highly central node is a carrier (tick/vertebrate) found to be infected by many cargo haplotypes that infect many other carriers in the network. The vertebrates or the ticks with the greatest centrality are super-spreaders [59].

We aimed to obtain measures of the phylogenetic diversity of the *A. phagocytophilum* haplotypes at the level of species of vertebrates and ticks. These calculations are intended to understand if a higher or lower phylogenetic diversity of *A. phagocytophilum* is linked to some groups of vertebrates/ticks. In other words, we aimed to explicitly demonstrate the association of given haplotypes or their clusters to species of vertebrates or ticks. The phylogenetic tree of haplotypes of *A. phagocytophilum* implicitly includes a measure of distance among the tips of the tree. Phylogenetic diversity of *A. phagocytophilum* per vertebrate/tick species was calculated using Faith’s phylogenetic diversity (PD) [60] as the total branch length spanned by the tree, including all of the haplotypes of *A. phagocytophilum* recorded in a single species of “carrier”. We also calculated the mean pairwise distance (MPD) as described previously [61]. Null models were generated that randomized the tips of the phylogeny to calculate the significance of the phylogenetic association between ticks/vertebrates and haplotypes of *A. phagocytophilum* evaluating the significance of MPD [61]. The package *picante* [62] of the R programming environment [57] was used for these calculations. Both PD and MPD aim to demonstrate the significance of associations with portions of the phylogenetic tree of the haplotypes of *A. phagocytophilum* using different methods. The congruence of results between methods is supportive of such phylogenetic clustering.

## Results

### Haplotypes of *A. phagocytophilum* cluster along vertebrate, tick and geographical gradients

**Fig. 1:**
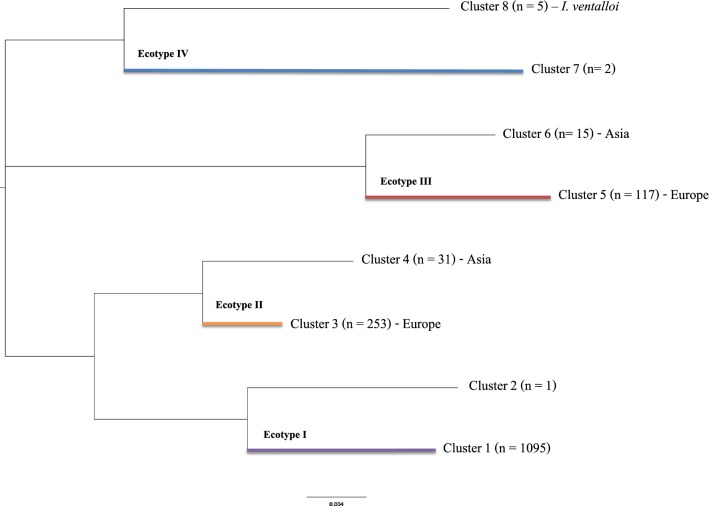
The phylogenetic tree of the clusters of *A. phagocytophilum* haplotypes detected in this study. Each branch of the tree includes the name of the cluster, with the number of haplotypes that belong to it, and some details of distribution or new carriers. Coloured lines labelled with the “Ecotype” refer to the original groups proposed by Jahfari et al. [31]

We identified 199 unique haplotypes in a total of 1623 isolates of *A. phagocytophilum*. Tables 1 and 2 summarize the field isolates according to vertebrates and questing or feeding vectors, respectively. In 730 vertebrate isolates, 135 *A. phagocytophilum* haplotypes were recorded, while 127 haplotypes were detected in 893 ticks: 789 collected from the vegetation and 104 feeding on vertebrates (Tables 1, 2). Haplotypes were grouped into 8 clusters according to the phylogenetic tree using shorter DNA fragments from 1992 isolates (Fig. 1, Additional file 1: Table S1). However, a network analysis of the relationships among haplotypes, ticks and vertebrates (see below) produced a total of 12 communities of interacting organisms. All of these extra-communities, containing only one or a few haplotypes, remained undetected in the phylogenetic tree. We chose to keep the original numbering of clusters based on genetic procedures (i.e. 8 clusters), while retaining the complete structure of the network to show the relationships among interacting partners.

**Table 1:** List of vertebrates, divided by taxonomic orders (columns) and countries of collection, that yielded a 530-bp *groEl* fragment of *A. phagocytophilum.* The number of haplotypes and clusters per vertebrate order is shown

Country	Artiodactyla	Aves	Carnivora	Erinaceomorpha	Primates	Lagomorpha	Perissodactyla	Rodentia	Soricomorpha	Total
Albania	1		3							4
Austria	22				1					23
Belgium			3		1					4
Brazil			12							12
Czech Republic	1	3		11				1		16
Finland			2							2
France	27						3			30
Germany	52		6	2			15			75
Hungary			1	54						55
Italy			7				3	4		14
Netherlands	149		7				13	5		174
Norway	105							22	63	190
Poland	9		1					1		11
Scotland	2									2
Slovakia	5							5		10
Slovenia	29		5		1					35
Spain	3									3
Sweden	2						2	7		11
Switzerland	8						1	1		10
Japan	3		1							4
Korea	1		3		1			5		10
Russia								11	1	12
USA			3		4	1	7	8		23
Total	419	3	54	67	8	1	44	70	64	730
Haplotypes	106	2	16	3	4	1	15	15	2	
Clusters	4	2	2	1	2	1	1	4	1

**Table 2:** List of arthropod samples collected from the vegetation and from vertebrates that yielded a 530-bp *groEl* fragment of *A. phagocytophilum.* Included are the number of haplotypes

Arthropod species	Sampling on	Samples (*n*)	Haplotypes (*n*)
*Dermacentor reticulatus*	Vegetation	1	1
*Haemaphysalis douglasi*	Vegetation	1	1
*Haemaphysalis flava*	*Hydropotes inermis*	2	1
*Ixodes acuminatus*	Vegetation	3	1
*Ixodes frontalis*	Aves	2	1
*Ixodes hexagonus*	Vegetation	1	1
	*Erinaceus europaeus*	22	1
	*Martes martes*	2	1
	*Mustela putorius*	4	3
*Ixodes nipponensis*	Vegetation	1	1
	*Hydropotes inermis*	2	1
*Ixodes pacificus*	Vegetation	2	2
*Ixodes pavlovskyi*	Vegetation	3	2
*Ixodes persulcatus*	Vegetation	26	7
	*Hydropotes inermis*	1	1
	Rodentia	3	1
*Ixodes ricinus*	Vegetation	741	101
	Aves	6	1
	*Capreolus capreolus*	14	8
	*Cervus elaphus*	5	5
	Deer (unknown species)	4	2
	*Erinaceus europaeus*	7	1
	*Homo sapiens*	3	3
	*Mustela erminea*	2	2
	*Martes martes*	8	3
	*Ovis aries*	1	1
	Rodentia	3	3
*Ixodes trianguliceps*	Vegetation	4	4
	Rodentia	6	3
*Ixodes ventalloi*	Vegetation	6	3
*Lipoptena cervi*	*Cervus elaphus*	6	5
*Rhipicephalus sanguineus* (*s.l.*)	*Canis familiaris*	1	1
Total		893

### Association of *A. phagocytophilum* haplotypes to countries, hosts and vectors

**Fig. 2:**
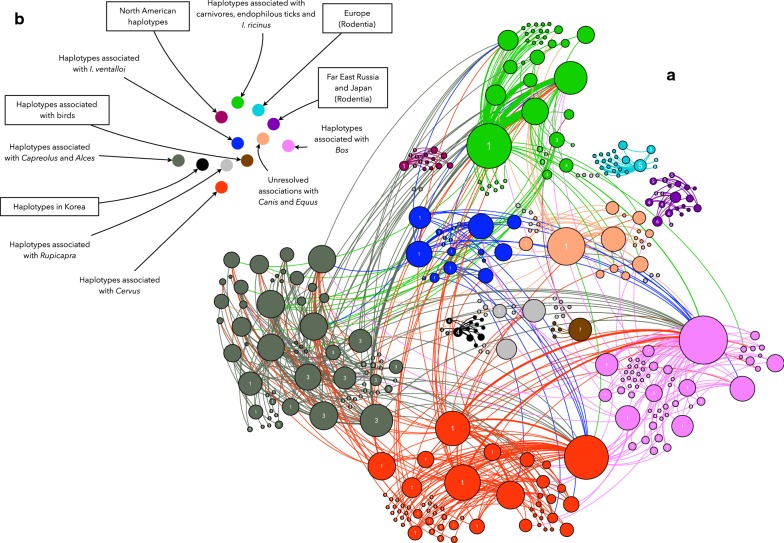
**a** The network of the communities of *A. phagocytophilum* haplotypes, as linked to carriers (ticks or vertebrates). Circles (nodes) are haplotypes from ticks or vertebrates, but the labels are included only in nodes representing haplotypes of *A. phagocytophilum* to improve reading (i.e. labels are not included for ticks and vertebrates). Labels are the number of the cluster obtained in the phylogenetic tree in Fig. 1. The colours represent the communities detected by an agglomerative clustering algorithm and the size of each circle is proportional to its centrality in the network. Large circles are organisms that are well represented in the network (i.e. a carrier in which several haplotypes have been detected, or a haplotype that widely circulates among different carriers). The links among nodes represent relationships among interacting organisms (a haplotype and a carrier) displaying the colour of the community. The width of the link is proportional to the number of interactions among the organisms. The complete network with labels for every node is included in Additional file 2: Figure S1. **b** A reduced version of the network is included to simplify interpretations. Each circle has the same colour as the set of nodes of each cluster in a. Labels identify the communities that do not necessarily match the phylogenetic clusters. Labels enclosed in rectangles are communities that are disconnected from the giant component of the network

*Anaplasma phagocytophilum* haplotypes were linked to the carriers (hosts and vectors) and countries in which they were recorded, resulting in a network (Fig. 2 and Additional file 2: Figure S1). The haplotypes linked to Cluster 1 were present in the widest range of carriers: Artiodactyla, Perissodactyla, Carnivora, Nearctic Rodentia, *I. ricinus*, *I. scapularis*, *I. pacificus* and *I. hexagonus*. Cluster 1 remained unrecorded in six other species of ticks. Geographically, Cluster 1 was present in the Western Palaearctic and Nearctic regions (Fig. 2, Additional file 3: Figure S2). However, Cluster 1 was found to be absent in rodents in Far Eastern Russia, Japan and Korea. Cluster 4 is a segregated group of haplotypes recorded in South Korea, *Ixodes persulcatus* ticks in Russia and one vertebrate in Japan. Cluster 5 appeared in rodents and *I. trianguliceps* in Europe. Cluster 6 was recorded in rodents or rodent-feeding ticks (*I. persulcatus*) in Japan. Haplotypes similar to those in Cluster 5 were also detected in the group of haplotypes restricted to Far East Russia and Japan. Cluster 7 is a group of haplotypes associated with birds and endophilous ticks associated with them (such as *I. frontalis*).

The network of interacting organisms (Fig. 2) is a tightly nested set of relationships. The giant component of the network (i.e. the one that stays connected) had 117 articulation points. This means a high resilience of the network against the removal of one of these articulation points, allowing ample circulation of the bacterium. Cluster 1, Cluster 2 (detected in *Capra aegagrus*, *Ovis aries* and *Rupicapra rupicapra*) and Cluster 3 were heavily linked to *Ixodes ricinus*, highlighting the dominance of this vector in the circulation of most *A. phagocytophilum* haplotypes in Europe (Fig. 2). The remaining clusters 4 to 8 were either isolated or can be disconnected from the giant component by removing only one articulation point. This finding means that every haplotype recorded in rodents in Europe is independent of the main circulation of other haplotypes in the network. Additionally, this implies that these haplotypes are different from those recorded in rodents and associated ticks in Russia and Japan, but still retain some genetic similarities with haplotypes circulating among rodents in Europe. Haplotypes recorded in South Korea were also independent of the complete network. The disconnection of this group of South Korean haplotypes seems to be derived from the fact that they are associated with different tick species (*Haemaphysalis* spp.). This finding must be considered with caution since it was impossible to know if *A. phagocytophilum* is circulated by ticks of the genus *Haemaphysalis*, because every tick was collected while feeding. Nevertheless, these associations promote a disconnection of these haplotypes with others found in Japan and Far East Russia. Additionally, the haplotypes recorded from birds and engorged *I. frontalis* (Cluster 7) were disconnected from the main giant network component, even if there was an overlapping geographical distribution with other hosts and *I. ricinus* (Fig. 2). No clear conclusions can be drawn about the segregation of haplotypes connected to carnivores and their endophilous ticks (such as *I. hexagonus*) since they shaped a separate cluster but were also tightly connected to the rest of the giant component (Fig. 2 and Additional file 2: Figure S1). The isolates from North America formed a disconnected sub-network (Fig. 2) which resulted from the lack of geographical overlap with the European isolates. In any case, both European and American human isolates belong to Cluster 1. It is noteworthy that a previously unexplored species of tick, *I. ventalloi*, provided haplotypes belonging to a completely different cluster.

**Fig. 3:**
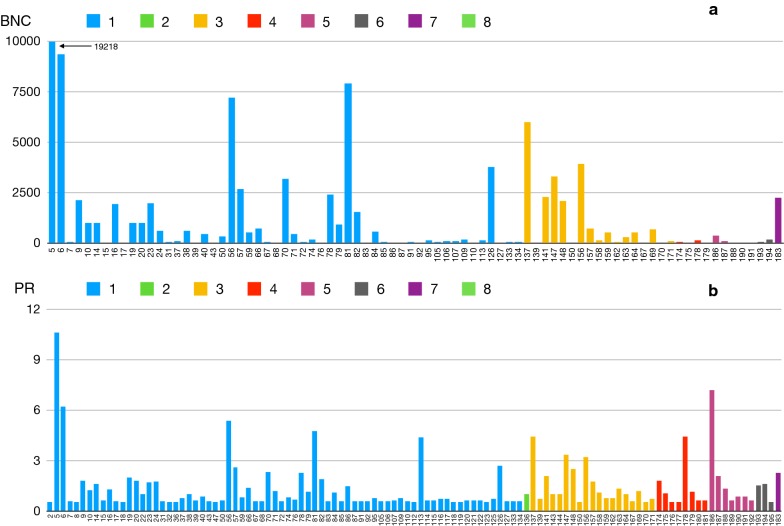
Values of betweenness centrality (BNC) (**a**) and PageRank (PR) (**b**) for each of the haplotypes of *A. phagocytophilum*. Only the haplotypes with values higher than 0 for either BNC or PR are included in each chart. Categories correspond to the phylogenetic clusters shown in Fig. 1. Betweenness centrality represents the relative importance of a haplotype in the complete network. Betweenness centrality is related to the connectivity of a network, in so much as high betweenness vertices have the potential to disconnect graphs if removed. Therefore, a node with high BNC is a node that is very central and connected to many other nodes. PR represents the importance of the nodes to which a given link is connected. It is thus a measure of the potential spread of a node through connected nodes, according to its relative importance

Figure 3 includes the values of BNC and PR for the haplotypes, grouped according to clusters. To simplify visualization, we included only haplotypes with a BNC higher than 0 or a PR higher than 0.5. A high BNC value is typical for a major hub in a network, “circulating” many haplotypes. For the complete *A. phagocytophilum* network, the major hub was *I. ricinus*, with BNC values in the range of 600–25,000 for the associated haplotypes. Other tick species scored much lower in BNC values: *I. ventalloi* (BNC = 1098); *I. persulcatus* (BNC = 264); *I. pavlovskyi* (BNC = 24); and *I. trianguliceps* (BNC = 14). Haplotypes with high PR values were connected to well-represented carriers in the network, therefore having larger probabilities for spreading. Both indices were only partially correlated. The correlation between values of BNC and PR was clear for haplotypes in clusters 1 and 3. However, while network indices indicate high values of PR for haplotypes in clusters 5 and 6 (restricted to rodents and geographically separated) and therefore high chances of spread, their low values of BNC confirm that they are poorly connected within the network. This is hypothesized to happen due to a lack of circulation out of the subnetwork(s) containing the vertebrates and ticks in which they have been recorded.

### Association of *A. phagocytophilum* haplotypes with vertebrate and tick species

To further demonstrate how the haplotypes fit into the framework of clusters, the association of vertebrates (Table 3) and (feeding) arthropods (Table 4) to certain clusters was tested with the Monte Carlo method. Confirming the results from the network analysis, a significant support was found for an association of *I. ricinus* with the large Cluster 1 (Table 4). An association of *I. hexagonus*, feeding on *Erinaceus europaeus*, with Cluster 1 was also found (Table 4). We found a significant association of *I. ricinus* feeding on *C. elaphus* with Cluster 1 (Table 4), and of *I. ricinus* feeding on *Capreolus capreolus* with Cluster 3. Cluster 4 was significantly linked to questing *I. persulcatus* ticks and *Apodemus agrarius*, a rodent found in eastern Europe and Asia. This cluster contained different samples, mainly from South Korea and Russia, and was not limited to Soricomorpha-Rodentia and *I. trianguliceps* like Cluster 5 (Europe) or to only Rodentia and *I. pavlovskyi/I. persulcatus* ticks like Cluster 6 (eastern Europe and Asia).

**Table 3:** Selective vector distributions between the clusters of *A. phagocytophilum* haplotypes (columns). Only vertebrate hosts with more than one isolate are shown. The Monte Carlo method was used to estimate the probability (*P*-value) that the number of most numerous clusters in a random realization from the multinomial is equal to or greater than the observed maximum among the isolates

Vertebrate species	1	2	3	4	5	6	7	8	Total (*n*)
*Alces alces*	23*		12						35
*Bos taurus*	29*								29
*Capra aegagrus*	4	1	3						8
*Capreolus capreolus*	10		95*						105
*Cervus elaphus*	105*								105
*Capra ibex*	2								2
*Cervus nippon*	5*								5
*Dama dama*	14*		1						15
*Ovis aries*	83*								83
*Ovis musimon*	16*								16
*Rupicapra rupicapra*	7*								7
*Sus scrofa*	5*								5
*Turdus merula*	2						1		3
*Canis familiaris*	32*			2					34
*Felis catus*	2			2					4
*Neotoma* sp.	7*								7
*Nictereutes procyonoides*	3								3
*Ursus arctos*	2								2
*Vulpes vulpes*	7*								7
*Mustela putorius*	2								2
*Erinaceus europaeus*	8*								8
*Erinaceus roumanicus*	59*								59
*Equus caballus*	44*								44
*Homo sapiens*	7*			1					8
*Myodes glareolus*				1	24*				25
Rodentia	1				15*				16
*Apodemus agrarius*				5*					5
*Myodes rufocanus*					2	2			4
*Myodes rutilus*					3	1			4
*Apodemus sylvaticus*					2				2
*Microtus agrestis*					3				3
*Tamias sibiricus*				2					2
*Sorex araneus*					30*				30
*Sorex isodon*					33*				33
No. per cluster	479	1	111	8	112	3	1	0	720

*Note*: A low *P*-value (*P *<  0.05) indicates a significant association between a cluster and the species of vertebrate (marked with an asterisk)

**Table 4:** Selective vector distributions between clusters of *A. phagocytophilum* haplotypes (columns). The Monte Carlo method was used to estimate the probability (*P*-value) that the number of most numerous clusters in a random realization from the multinomial is equal to or greater than the observed maximum among the isolates

Arthropod species	Sampling on	1	3	4	5	6	7	8	Total (*n*)
*Dermacentor reticulatus*	Vegetation					1			1
*Haemaphysalis douglasi*	Vegetation								1
*Haemaphysalis flava*	*Hydropotes inermis*			2					2
*Ixodes acuminatus*	Vegetation	3							3
*Ixodes frontalis*	Aves						2		2
*Ixodes hexagonus*	Vegetation	1							1
	*Erinaceus europaeus*	22*							22
	*Martes martes*	2							2
	*Mustela putorius*	3	1						4
*Ixodes nipponensis*	Vegetation			1					1
*I. nipponensis*	*H. inermis*			2					2
*Ixodes pacificus*	Vegetation	2							2
*Ixodes pavlovskyi*	Vegetation			1		2			3
*Ixodes persulcatus*	Vegetation			16*		10			26
	*H. inermis*			1					1
	Rodentia					3			3
*Ixodes ricinus*	Vegetation	601*	139				1		741
	Aves						6*		6
	*Capreolus capreolus*	1	13*						14
	*Cervus elaphus*	5*							5
	Deer (unknown species)	1	3						4
	*Erinaceus europaeus*	7*							7
	*Homo sapiens*		3						3
	*Mustela erminea*	1	1						2
	*Martes martes*	7*	1						8
	*Ovis aries*		1						1
	Rodentia	1	2						3
*Ixodes trianguliceps*	Vegetation				4*				4
	Rodentia				6*				6
*Ixodes ventalloi*	Vegetation	1						5*	6
*Lipoptena cervi*	*Cervus elaphus*	3	3						6
*Rhipicephalus sanguineus* (*s.l.*)	*Canis familiaris*	1							1
No. per cluster		662	167	23	10	16	9	5	893

*Note*: A low *P*-value (*P* < 0.05) indicates a significant association between a cluster and the species of (engorging) arthropod (marked with an asterisk)

Table 5 includes the values of PD for the species of vertebrates for which samples of *A. phagocytophilum* were obtained. A low PD value means phylogenetic clustering and therefore a lower variability in the samples associated with a given vertebrate. Eighteen species of vertebrates showed values of significant association with a given portion of the phylogenetic tree of *A. phagocytophilum*. The same index was provided for the arthropods (ticks and one species of insect, *Lipoptena cervi*) with similar results for *I. pacificus*, *I. ricinus* and *I. trianguliceps* (Table 6). Samples recorded in *I. ricinus* were separately evaluated for PD according to the geographical origin, resulting in highly significant associations with given haplotypes of *A. phagocytophilum*. If data from *I. ricinus* were included in calculations without geographical segregation, the PD value for the tick was 0.94 (highly non-significant). At least in the case of the most prominent tick vector, the associations of *A. phagocytophilum* with *I. ricinus* are of a local nature: considering these associations at a continental level could not give evidence to the segregation of haplotypes in the tick, strongly suggesting an adaptation to regional populations of the tick. Tables 7 and 8 include further results about phylogenetic clustering using the mean pairwise genetic distance, comparing the observed phylogenetic relatedness of the haplotypes recorded for vertebrates/arthropods to the pattern expected under a null model of phylogeny or community randomization. Results confirm the findings obtained by PD: there is a phylogenetic clustering of the haplotypes of *A. phagocytophilum* with the carriers. The highly significant values for *I. ricinus* and *I. trianguliceps* are demonstrative of segregation of the bacterium in these ticks, further supported by the significant values found in *I. pacificus*, a tick with a distribution range disconnected from the main range of the others.

**Table 5:** Faith’s phylogenetic diversity (PD) of the *A. phagocytophilum* haplotypes associated to each vertebrate species investigated. A low PD value indicates a significant association between a haplotype and a vertebrate (marked with an asterisk)

Vertebrate order	Vertebrate species	Samples (*n*)	Haplotypes (*n*)	PD
Artiodactyla	*Alces alces*	35	22	0.10
	*Bos taurus*	29	15	0.01*
	*Capra aegagrus*	8	7	0.36
	*Capreolus capreolus*	105	33	0.01*
	*Cervus elaphus*	105	29	0.01*
	*Cervus nippon*	7	7	0.41
	*Dama dama*	15	8	0.12
	*Felis catus*	4	4	0.54
	*Ovis aries*	83	19	0.01*
	*Ovis musimon*	16	11	0.02*
	*Rupicapra rupicapra*	7	7	0.02*
	*Sus scrofa*	5	3	0.01*
Aves	*Turdus merula*	3	2	0.94
Carnivora	*Canis familiaris*	34	13	0.05*
	*Mustela putorius*	2	2	0.05*
	*Neotoma* sp.	7	3	0.01*
	*Ursus arctos*	2	2	0.32
	*Vulpes vulpes*	7	2	0.02*
Erinaceomorpha	*Erinaceus europaeus*	8	2	0.01*
	*Erinaceus roumanicus*	59	2	0.04*
Perissodactyla	*Equus caballus*	44	15	0.01*
Primates	*Homo sapiens*	8	4	0.46
Rodentia	*Apodemus agrarius*	5	3	0.01*
	*Myodes glareolus*	25	3	0.38
	*Myodes rufocanus*	4	3	0.01*
	*Myodes rutilus*	4	2	0.05*
Soricomorpha	*Sorex araneus*	30	2	0.01*

**Table 6:** Faith’s phylogenetic diversity (PD) of the *A. phagocytophilum* haplotypes associated with the investigated arthropods species. A low PD value indicates a significant association between a haplotype and an arthropod species (marked with an asterisk)

Arthropod (species)	Samples (*n*)	Haplotypes (*n*)	PD
*Ixodes pacificus*	2	2	0.01*
*Ixodes pavlovskyi*	3	2	0.81
*Ixodes persulcatus*	26	7	0.13
*Ixodes ricinus*	741	101	0.01*
*Ixodes trianguliceps*	4	4	0.01*
*Ixodes ventalloi*	6	3	0.46
*Lipoptena cervi*	6	5	0.20

*Notes*: The *I. ricinus* isolates were first separated according to the 17 countries of collection. Four countries (Germany, the Netherlands, Norway and Slovakia) represent 83% of the *I. ricinus* isolates. Results for *I. ricinus* are the average of each sample/country calculated separately

**Table 7:** The mean pairwise distance (MPD) of *A. phagocytophilum* haplotypes found in different vertebrate species. MPD-C is the comparison of MPD against null communities

Species	No. of haplotypes	MPD	MPD-C	MPD (*P*-value)
*Alces alces*	22	1.19	− 1.20	0.12
*Bos taurus*	15	0.62	− 5.71	0.01*
*Capra aegagrus*	7	1.35	0.03	0.49
*Capreolus capreolus*	33	0.91	− 7.44	0.01*
*Cervus elaphus*	29	0.96	− 5.59	0.01*
*Cervus nippon*	7	1.32	− 0.04	0.45
*Dama dama*	8	1.03	− 1.64	0.08
*Ovis aries*	19	0.77	− 5.50	0.01*
*Ovis musimon*	11	0.72	− 4.39	0.01*
*Rupicapra rupicapra*	7	0.79	− 2.69	0.02*
*Sus scrofa*	3	0.32	− 3.11	0.01*
*Turdus merula*	2	1.96	1.00	0.83
*Canis familiaris*	13	1.08	− 2.42	0.03*
*Felis catus*	4	1.43	0.24	0.59
*Mustela putorius*	2	0.11	− 2.02	0.04*
*Ursus arctos*	2	1.01	− 0.52	0.22
*Vulpes vulpes*	2	0.11	− 1.92	0.05*
*Erinaceus europaeus*	2	0.07	− 2.38	0.03*
*Erinaceus roumanicus*	2	0.07	− 2.21	0.03*
*Equus caballus*	15	0.92	− 3.47	0.02*
*Homo sapiens*	4	1.40	0.25	0.52
*Apodemus agrarius*	3	0.02	− 3.87	0.01*
*Myodes glareolus*	3	1.32	− 0.13	0.33
*Myodes rufocanus*	3	0.06	− 3.12	0.01*
*Myodes rutilus*	2	0.09	− 2.07	0.04*
*Neotoma* sp.	3	0.09	− 3.31	0.01*
*Sorex araneus*	2	0.04	− 2.24	0.01*

*Notes*: Negative values of MPD-C together with low *P*-values mean significant associations of the haplotypes to the species of vertebrate (marked with an asterisk)

**Table 8:** The mean pairwise distance (MPD) of *A. phagocytophilum* haplotypes found in different arthropod species. MPD-C is the comparison of MPD against null communities

Arthropod (species)	Haplotypes (*n*)	MPD	MPD-C	MPD (*P*-value)
*Ixodes pacificus*	2	0.11	− 2.12	0.04*
*Ixodes pavlovskyi*	2	1.96	0.89	0.82
*Ixodes persulcatus*	7	1.13	− 1.26	0.11
*Ixodes ricinus*	101	1.15	− 5.66	0.01*
*Ixodes trianguliceps*	4	0.05	− 4.31	0.01*
*Ixodes ventalloi*	3	1.31	− 0.20	0.37
*Lipoptena cervi*	5	1.21	− 0.59	0.23

*Notes*: Negative values of MPD-C together with low *P*-values mean significant associations of the haplotypes to the species of arthropod (marked with an asterisk)

## Discussion

Enhancing the understanding of the transmission networks of tick-borne pathogens requires a challenging blend of molecular biology, phylogenetics and network theory. Here, we delineated the existence of a relatively high number of haplotypes of *A. phagocytophilum* by standard molecular methods, based on the presence of DNA and bacterial *groEL* sequences originating from a large variety of ticks and vertebrates from a broad geographical range. These haplotypes grouped into eight well-defined genetic clusters. We acknowledge that presence of bacterial DNA in samples of vertebrates or ticks is not sufficient to document the infectivity of the bacterium. In addition, vector competence requires ticks to be infected and passing viable pathogens to the next stage and to a new host. We also recognize that the sampling effort has not been the same for every region, vertebrate or tick. This is, however, currently the most geographically extensive and largest available dataset of *groEL* sequences of *A. phagocytophilum*. It provides an unexpected picture of the considerable spread of some haplotypes and the affinity of some others towards well-defined groups of vertebrates, ticks and regions, expanding the previous view on the evolution and segregation of the bacterium [15, 21, 28, 31].

### Reservoir-driven rather than vector-driven selection mechanisms?

This study corroborated the preferential and differential association of lineages of the bacterium to several groups of vertebrates and ticks, also showing a geographical variation in pathogen-host-vector associations. Observed delineation of *A*. *phagocytophilum* could be driven by geographical isolation (environmental traits acting on ticks or reservoirs), or by peculiarities of the life-cycle or interacting molecular phenomena of ticks and/or reservoirs, including immune peculiarities differing across hosts. The different geographical distribution of some groups of Artiodactyla in which *A. phagocytophilum* has been recorded is a support for a geographical isolation. However, it has been demonstrated that haplotypes circulating in *Cervus elaphus* are different from those associated with *Alces alces*, even if some mix of haplotypes can be found in both vertebrates because of habitat overlap and a shared tick vector being the same (*I. ricinus*) [41]. This provides support for reservoir-driven delineation and appears to exclude a vector-driven selection mechanism of haplotypes. Reservoir-driven delineation is also supported by the finding that the cluster of haplotypes associated with birds is separated from the main set of haplotypes circulating through mammals. While birds are prominent hosts for the immature stages of *I. ricinus* [63] they do not seem competent hosts of the main mammals-associated haplotypes, and birds could filter these haplotypes and prevent their spread through birds carrying *I. ricinus*. This is an interesting outcome of our study, since only a few (4 out of 857) *A. phagocytophilum*-positive *I. ricinus* from the vegetation and mammals were infected with haplotypes associated with birds [64], likely due to a tick strictly specific to birds, *I. frontalis*, being vector of the bird-associated haplotypes. This would contribute to the restricted circulation of most mammal-associated haplotypes through birds carrying *I. ricinus*.

### Geographical differences in haplotype clustering

Previous studies demonstrated the potential of networks to disentangle the complex relationships among ticks, transmitted pathogens and vertebrates in general [22] and the tight association between strains of *Borrelia burgdorferi* (*s.l.*) and allopatric species of ticks [58]. The former study set the basics for unravelling these ecological associations, the later outlined the segregation of *B. burgdorferi* (*s.l.*) according to the species of vector ticks. These general insights into networks of ticks, pathogens and hosts were also found in *A. phagocytophilum*: considered as a single species, it seems to evolve along different lines that shape clusters of haplotypes that are, in some cases, tightly circumscribed to particular combinations of vertebrates and ticks. The combined use of both molecular and network-derived methods pointed out that rodents have at least two different clusters of haplotypes. The first one, already outlined [31], is linked with rodents in the Western Palaearctic, while the other cluster is linked with rodents (and ticks feeding on rodents) in the Far Eastern Russia and Japan. Of most importance, the present results from the networks point to a restricted circulation of these clusters, even when the ecological communities suggest they could have the potential for a wider circulation. Moreover, phylogenies clearly point to a close relationship between Cluster 5 (former ecotype III, rodents in Europe) and Cluster 6 (rodents plus feeding ticks in Far Eastern Russia and Japan), suggesting that the disconnection of these clusters could be derived from the gap in obtaining isolates in the large territory between Western and Eastern Russia. We hypothesize that these clusters associated with rodents and ticks of the *I. ricinus* complex could actually be a gradient of variability of strains of *A. phagocytophilum* throughout most of the Palaearctic, of which we captured “the picture” at both extremes.

It is striking that a high phylogenetic clustering of *A. phagocytophilum* in *I. ricinus* has been found only when the geographical component has been considered in calculations. This result strongly points to a separation of haplotypes not only regarding specific combinations of ticks and vertebrates, but also to a selection by an environmental gradient shaping the life-cycle of tick vectors, at least in the widespread *I. ricinus*. Since ticks are ectothermic organisms, climate strongly regulates their development and mortality rates, therefore resulting in populations of the tick exhibiting adaptations to a specific set of weather conditions [65]. A further selection of haplotypes of *A. phagocytophilum* by this mechanism, emerging as definite associations among haplotypes and populations of the tick, should not be discarded, since it has been already demonstrated for *Anaplasma marginale* [66]. An explicit examination of the effects of the environment, such as temperature and humidity, on the phylogenetic diversity of circulating haplotypes in ticks could not be addressed in this study because of the lack of coordinates of the isolates. The handling of the effects of environmental diversity assuming large areas like countries is not possible because of the considerable variety of environmental traits that can exist within a single geographical category.

### The role of other tick species

Other outcomes of this study provided unexpected relationships between *A. phagocytophilum*, vertebrates and other species of ticks. The largely neglected *I. ventalloi* has already been pointed out as carrier of *A. phagocytophilum* [67] but our results suggest a dual role for this tick regarding the circulation of the bacterium. Available data outlined that this tick species is involved in both a subnetwork of haplotypes with restricted circulation, serving as an articulation point with the giant component of haplotypes of the Cluster 1, and thus directly connected with haplotypes circulated by *I. ricinus*. This seems to derive from the large spectrum of hosts of *I. ventalloi* (formerly considered to be specific to the European rabbit, *Oryctolagus cuniculus*) and its sympatry with *I. ricinus* in its southern European range [68]. Interestingly, the cluster of haplotypes associated with *I. ventalloi* is more divergent than the Cluster 1, circulated by *I. ricinus*, which suggest a secondary evolution of the bacterium in the niche of *I. ventalloi* and its hosts. The results obtained for *I. ventalloi* support the finding that this tick constitutes an articulation point in the evolutionary pressures of haplotypes in the Clusters 1 and 8, the latter so far found exclusively in this tick. Both *I. ricinus* and *I. ventalloi* overlap in portions of their environmental niche in the southern portion of the distribution range of the former and share some hosts, the latter observing a semi-endophilic behaviour. It is thus not unexpected that a different cluster of haplotypes segregated in *I. ventalloi* because of its different life habits and environmental niche.

### Future studies and remaining challenges

Our data on the phylogenetic segregation of haplotypes of *A. phagocytophilum* in ticks revealed interesting results and potential for future studies. Of these, the most obvious is the high variability of haplotypes (low phylogenetic clustering) found in *I. persulcatus*, *I. pavlosvkyi* and *I. ventalloi*. *Ixodes pavlovskyi* exploits a portion of the environmental niche of *I. persulcatus* [58] and both are involved in the circulation of rodent haplotypes in Far Eastern Russia and Japan. This suggests the need of wide surveys collected in the gap between eastern Europe and Japan, incorporating new information to the network-derived construct, opening additional perspectives to the view presented here, and contributing to a better understanding of the evolution of such a singular bacterium.

A high clustering indicates a higher affinity of some haplotypes for a carrier than expected by chance, and therefore a segregation of haplotypes according to carriers, which would circulate different haplotypes. Our analysis revealed a higher than expected clustering of haplotypes of *A. phagocytophilum* for most species of vertebrates or ticks, with a few exceptions, such as the common blackbird *Turdus merula*, or the wild goat *C. aegagrus*. One could argue that the high values of phylogenetic clustering of *A. phagocytophilum* in vertebrates/ticks could be derived from the low sample sizes for some taxa, which is likely for some of our isolates. However, high values of phylogenetic clustering were also obtained with well-represented vertebrates, such as *C. elaphus*, *C. capreolus* or *Myodes* spp. The same is applicable to the ticks *I. ricinus* and *I. trianguliceps*, the former exceptionally well surveyed, the latter under-represented. Therefore, we contemplate these results reflect true phylogenetic relationships between the bacterium and either vertebrate or vector (or both). *Anaplasma phagocytophilum* has to survive in and colonize both a vertebrate and an arthropod, a dual mode of life, and therefore the still not completely studied metabolic adaptations of the bacterium need to cope with a large gradient of conditions regarding the defensive mechanisms of both partners. It is thus expected that the adaptive plasticity of the bacterium can be detected as a result of its fitness to particular conditions. Empirical studies exist about the tight interactions between the tick and the pathogen, demonstrating that *A. phagocytophilum* is able to rewire literally dozens of the tick metabolic pathways [69] and that infected ticks have an increased expression of some proteins, such as heat-shock proteins [70], allowing a prolonged survival under otherwise adverse environmental conditions. This kind of molecular adaptation has led to the ubiquitous occurrence of this bacterium both geographically and in terms of vertebrate and arthropod hosts which is an example of ecological fitting [71] that will enable its further spread. Studies from samples collected in the still unsurveyed gap would incorporate new information to the network-derived construct. Other than rodents, the role of birds in segregation of *A. phagocytophilum* should be further investigated, opening additional perspectives to the view presented here, and contributing to a well-focused overview of the evolution of such a singular bacterium.

## Conclusions

Our study incorporated methods from different fields, aiming to uncover the variability of haplotypes of *A. phagocytophilum* using the largest available set of isolates from ticks and vertebrates in the widest geographical area so far. The use of molecular methods, phylogenetic clustering and network-based associations of interacting organisms provided results about an unexpected molecular variability in the *groEL* gene of *A. phagocytophilum*. Results widely exceeded the previous view of the evolution of the bacterium, recording 199 haplotypes. These haplotypes conform so far to eight clearly separated clusters, either widely circulating in the Western Palaearctic, confined to specific taxa of vertebrates, or restricted to smaller regions and patently segregated from the main network. While *I. ricinus* is still unquestionably the driving force behind the circulation of most haplotypes, evidence is accumulating about the role of other ticks (*I. ventalloi*, *I. persulcatus*, *I. pavlovskyi*, *I. frontalis* and *I. trianguliceps*) circulating different clusters that remain unconnected with the main giant component of the network. Our data also suggest that the two clusters of haplotypes associated with rodents could actually be the extremes of a large gradient of evolving strains of the bacterium.

## Additional files

**Additional file 1: Table S1.** The dataset including all haplotypes recorded in this study, with details on the source of isolate (tick or vertebrate), the phylogenetic cluster in which it is classified and the complete sequence of the fragment.

**Additional file 2: Figure S1.** The complete network of haplotypes of *A. phagocytophilum* with labels for every node, including the number of haplotype, carriers (ticks or vertebrates) and geographical origin. Symbols and colours in the network are identical to those in Fig. 2.

**Additional file 3: Figure S2.** The geographical distribution (countries) recorded so far for the phylogenetic clusters of haplotypes of *A. phagocytophilum*.

## Data Availability

Data supporting the conclusions of this article are included within the article and its additional files. The complete dataset of haplotypes is provided in Additional file 1. Representative sequences generated in this study were submitted to the GenBank database under the Accession Numbers MN093151–MN093291.

## Abbreviations

rRNA: Ribosomal RNA
qPCR: Quantitative polymerase chain reaction
BNC: Betweenness Centrality
PR: PageRank
PD: Faith’s phylogenetic diversity
MPD: mean pairwise distance
