# Supplementary figures and images for: Anaplasma phagocytophilum evolves in geographical and biotic niches of vertebrates and ticks
Source: Parasit Vectors. 2019 Jun 28;12:328. doi: 10.1186/s13071-019-3583-8 (PMC6599317; doi:10.1186/s13071-019-3583-8)

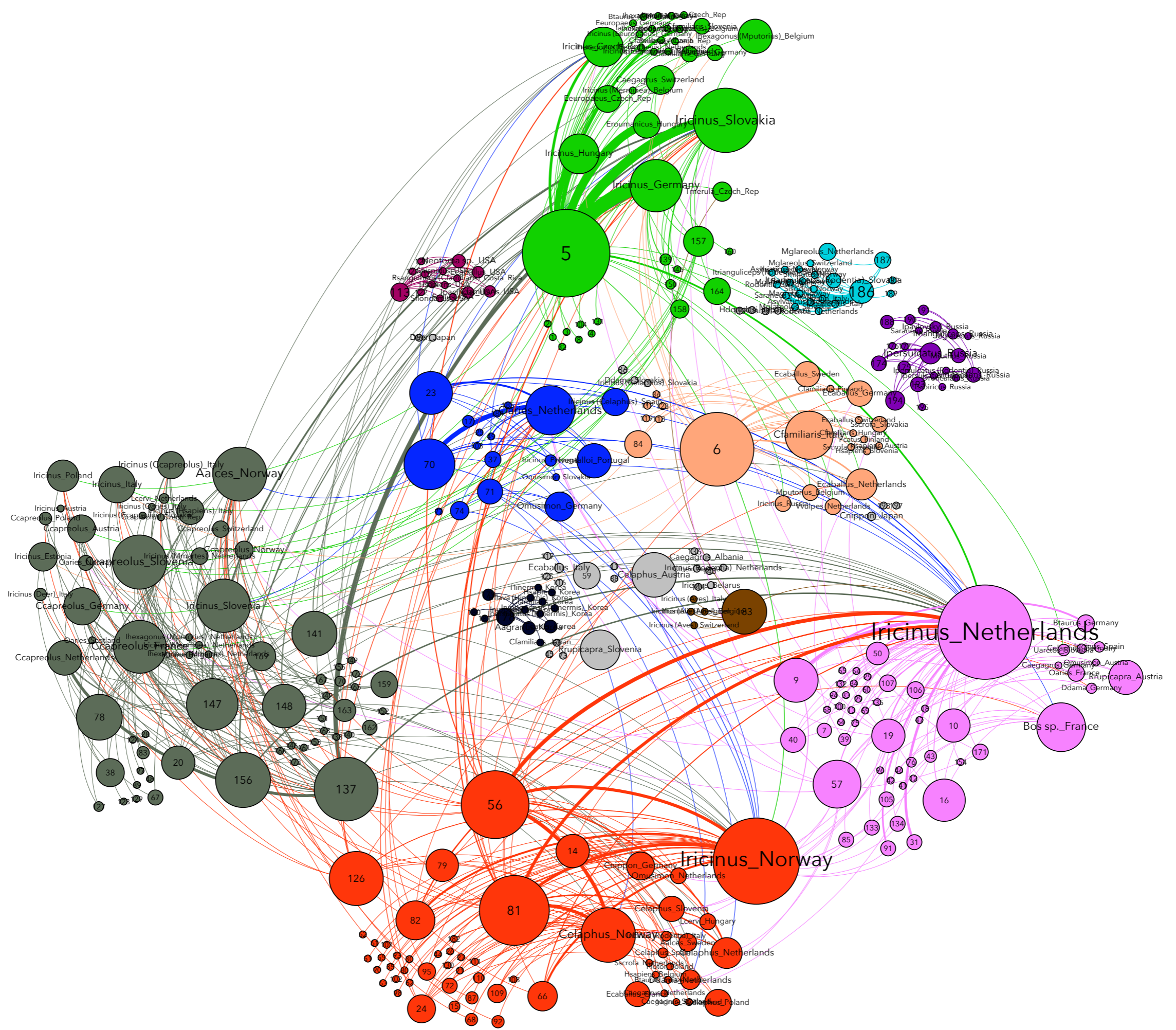

Supplement: Supplementary file 2 — Additional file 2: Figure S1. The complete network of haplotypes of A. phagocytophilum with labels for every node, including the number of haplotype, carriers (ticks or vertebrates) and geographical origin. Symbols and colours in the network are identical to those in Fig. 2. [file 13071_2019_3583_MOESM2_ESM.pdf]

Cluster 1

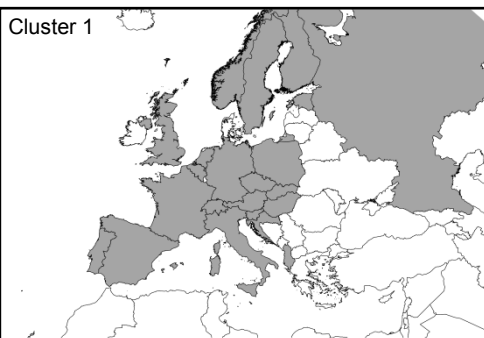

Cluster 2

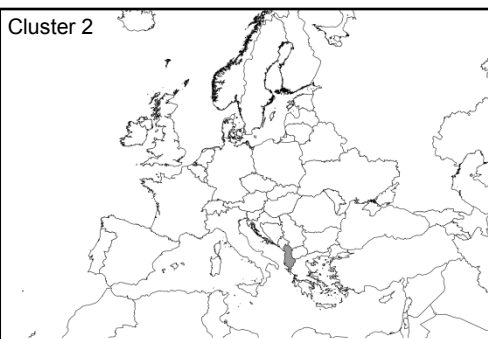

Cluster 3

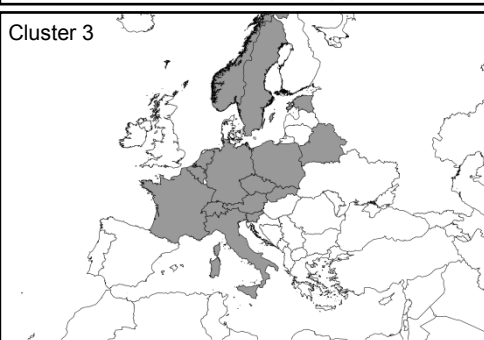

Cluster 4

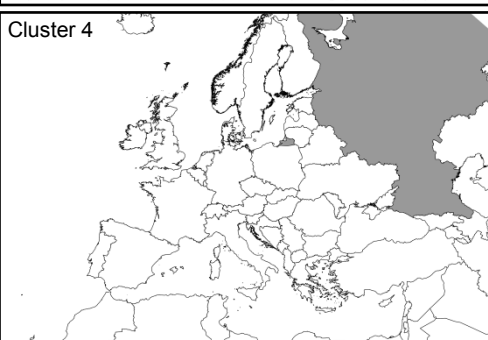

Cluster 5

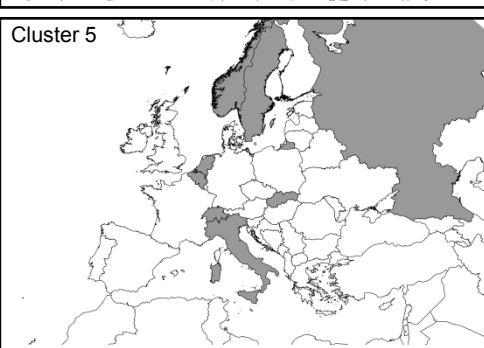

Cluster 6

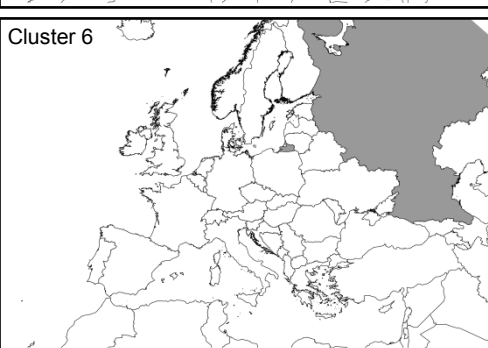

Cluster 7

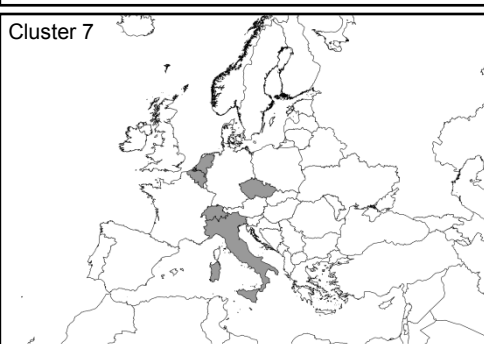

Cluster 8

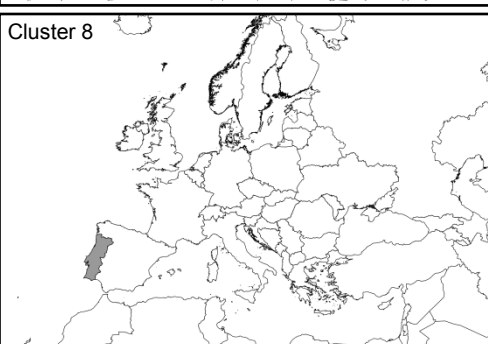

Supplement: Supplementary file 3 — Additional file 3: Figure S2. The geographical distribution (countries) recorded so far for the phylogenetic clusters of haplotypes of A. phagocytophilum. [file 13071_2019_3583_MOESM3_ESM.pdf]
